# Time and Angle-Resolved Time-of-Flight Electron Spectroscopy for Functional Materials Science

**DOI:** 10.3390/molecules27248833

**Published:** 2022-12-13

**Authors:** Nomi Lucia Ada Nathalie Sorgenfrei, Erika Giangrisostomi, Danilo Kühn, Ruslan Ovsyannikov, Alexander Föhlisch

**Affiliations:** 1 Institut für Methoden und Instrumentierung der Forschung mit Synchrotronstrahlung, Helmholtz-Zentrum Berlin für Materialien und Energie GmbH, Helmholtz-Zentrum Berlin GmbH, Albert-Einstein-Str. 15, 12489 Berlin, Germany; 2 Institut für Physik und Astronomie, Universität Potsdam, Karl-Liebknecht-Stra§e 24/25, 14476 Potsdam, Germany

**Keywords:** photoelectron spectroscopy, surface science, time-resolved, ultrafast, instrumentation, dichalcogenides, phase transition

## Abstract

Electron spectroscopy with the unprecedented transmission of angle-resolved time-of-flight detection, in combination with pulsed X-ray sources, brings new impetus to functional materials science. We showcase recent developments towards chemical sensitivity from electron spectroscopy for chemical analysis and structural information from photoelectron diffraction using the phase transition properties of 1T-TaS_2_. Our development platform is the *SurfaceDynamics* instrument located at the *Femtoslicing facility* at BESSY II, where femtosecond and picosecond X-ray pulses can be generated and extracted. The scientific potential is put into perspective to the current rapidly developing pulsed X-ray source capabilities from Lasers and Free-Electron Lasers.

## 1. Introduction

Functional materials characterization on the level of atomic constituents and their chemical state and interactions requires ideally the combined insight into the electronic structure, chemical state as well as structural features. Functionality also includes a response to external stimuli, non-equilibrium states and dynamic evolution of the material. This ability to change state is typically linked to the high sensitivity of the material itself to seemingly small external drivers. Thus, any measurement process needs to be designed to create as little disturbance as possible, avoiding sample damage. In addition one may want to track temporal evolution following ultrafast excitations over many orders of magnitude in time. Dynamics start on the femtosecond time scale and continue via transient states to equilibrium. Electron Spectroscopy for Chemical Analysis (ESCA) and X-ray Photo Electron Diffraction (XPD) allow us to identify the chemical state and local order parameters of individual atoms within the functional material. While hemispherical analyzers excel in terms of resolution, they lack the full two-dimensional mapping of emitted electrons. For toroidal electron analyzers the situation is reversed. They excel in terms of acceptance angle but they have only moderate resolution. The angle resolved Time of Flight (ArTOF) detection scheme allows for both aspects—combining the highest detection efficiency with angular resolution in the opening angle and simultaneous acquisition of a sufficient range of photoelectron kinetic energies. ArTOFs rely on a time-based detection scheme that requires short X-ray radiation pulses from a Synchrotron, Laser or Free-Electron Laser (FEL) source with a repetition rate of up to a few MHz. Thus inherently, ESCA and XPD performed with an ArTOF have dynamic information multiplexed with angle and kinetic energy, once an optical pump pulse has been synchronized to the X-ray probe pulse. With this approach, the static and dynamic characterization of functional materials with regard to their electronic structure, chemical composition as well as local structure as they evolve can now be done at the lowest sample damage. In this work, we show how the *SurfaceDynamics* end station at BESSY II implements and spearheads these capabilities using synchronized femtosecond X-Ray pulses, showcasing TaS_2_. TaS_2_ is a layered material with covalent bonding within the layers and with weak van-der-Waals between the layers thus bridging the gap between adsorbed molecules on surfaces and a pure single crystal.

As discussed in [1], one must always strike a balance between space charge effects of simultaneously created electrons and the need to acquire spectral data of significant statistics in an acceptable time. The authors of [2,3,4], among others, discussed the effect of space charge in photoelectron spectroscopy. The repulsive interaction between emitted electrons can degrade the resolution. Optimum performance is given by single electron creation at the highest detection efficiency. The transmission of a hemispherical electron analyser is on the order of $10^{-3}$. In contrast, the time based ArTOF spectrometers have a transmission of approx. $10^{-1}$ [2,5,6,7]. In such a spectrometer, photoelectron energies and angles of emission are detected simultaneously and efficiently by an electrostatic lens system with a position and time resolving detector.

ESCA and XPD require photon energy in the soft X-ray regime while the time-resolved aspect demands for ultrashort pulses with suitable lengths. Considering those aspects, the available pulsed X-Ray sources all have their advantages and disadvantages. The strength of FELs is clearly the ultrashort pulse length on the order of 10 femtoseconds with tunable photon energy with access from the vacuum ultra-violet (VUV) over the soft to the hard X-Ray regime. However, they deliver ultrashort pulses with extremely high intensities. With photoelectron spectroscopy one rapidly suffers from space charge and the spectra are degraded in resolution [4,8]. Even with a reduced intensity, one has to extend the multi-hit capability of present detectors to efficiently use the slitless design of an ArTOF. Moreover, since the majority of FEL sources are using the self-amplified spontaneous emission (SASE) scheme they exhibit jitter in intensity, photon energy and time. On the other hand, laser-based higher harmonics generation (HHG) sources have no jitter in time but lack the tunability and access to photon energies in the soft X-Ray regime with reasonable intensity. In this manuscript we use an alternative approach in exploiting a practically jitter-free and tunable slicing source for femtosecond X-Ray pulses at reasonable intensities.

## 2. Materials and Methods

### 2.1. Extraction of Storage Ring Sub-Patterns

To enable the fully multiplexed ESCA, XPD and dynamic characterization by an ArTOF electron spectrometer, we implemented at the UE56/1-PGM beamline of BESSY II efficient strategies to extract and switch between a variety of pulse lengths and BESSY II storage ring sub-patterns, in order to match the X-ray pulse to the scientific needs given by the functional material under investigation. We use these capabilities here to illustrate the scientific benefits of the rapid development and deployment of high-performance pulsed X-ray sources.

**Figure 1:**
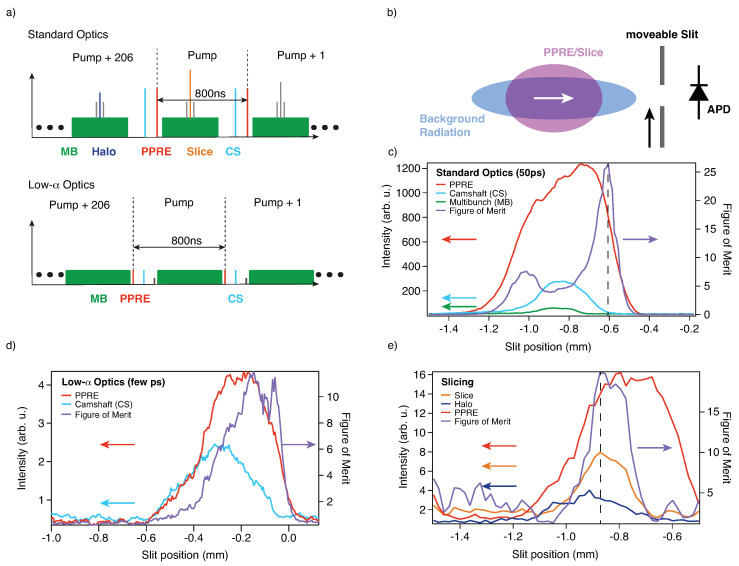
Extraction of sub-bunch patterns at BESSY II with different pulse lengths and repetition rates: (**a**) Sketch of the two fill patterns of BESSY II in the Surface Dynamics main chamber using the standard optics (50 ps FWHM) and low-α optics (few ps FWHM) of the storage ring. Multibunch (MB) in green, PPRE Bunch in red, fs-Slice Bunch in orange, Camshaft bunch (CS) in light blue, Slicing halo Background in dark blue (grey bunches are unused sliced bunches or the low current bunch on low-α operation). (**b**) Spatial filtering of sub-patterns in the spectral plane of the UE56/1-PGM beamline (the white arrow marks the direction of beam propagation). (**c**) Picosecond (50 ps FWHM) bunch extraction with 1.25 MHz repetition rate in PPRE operation (standard optics). (**d**) Picosecond (few ps FWHM) bunch extraction with 1.25 MHz repetition rate in PPRE operation (low-α optics). (**e**) Femtosecond (120 fs FWHM) bunch extraction with 6 kHz repetition rate in time slicing operation.

In Figure 1 we show how picosecond pulses with 1.25 MHz repetition rate and femtosecond pulses at 6 kHz are extracted using Pulse Picking by Resonant Excitation (PPRE) [9] and Femtoslicing [10] respectively at beamline UE56/1-PGM. These are two established approaches of bunch extraction in addition to mechanical choppers [11] and Transversal Island Buckets Filling (TRIBs) [12]. At a Synchrotron, the inherent picosecond time structure at MHz repetition rate can be used. At BESSY II, in particular, such pulses typically have a Full Width at Half at Maximum (FWHM) of about 50 ps in multibunch operation using the standard optics of the storage ring. Shorter bunches (down to a few ps) can also be produced using the so-called low-α mode. In low-α mode, the storage ring uses different optics and fill patterns, which allow for bunch lengths in the few ps regime [13]. For even shorter bunches the Synchrotron BESSY II provides at the Femtoslicing facility about 100 fs X-ray pulses with kHz repetition rate and flux on the order of $10^{6}$ photons/(s × 0.1% bandwidth) (Ref. [10] and references therein). Figure 1a illustrates the currently available fill patterns in multibunch and low-α operation modes.

The flux from the PPRE pulses is relatively low and the flux from the X-ray beam produced using slicing is even lower. Therefore, it is absolutely necessary to use a very high transmission electron spectrometer. The end station is provided with an Angular Resolved Time Of Flight electron analyzer (ARTOF). In our case we have installed one instrument with an acceptance angle of 60° [2,5,6,7]. The combination of short pulses from a coupled optical laser with short X-ray pulses enables the investigation of the dynamics of core levels.

The separation of the PPRE and slicing pulses is done in two steps. The first step is to detune the orbit of the electron bunches in the horizontal plane in a way such that the radiation of those bunches can be easily separated from unwanted multibunch radiation by beamline apertures. This is the so called *femtobump* [9,10]. The next step to further reduce the unwanted background radiation is to use a movable slit close to the focal point in the experimental chamber. The slit has a fixed width and is cut into a 100 μm thick quartz plate. The quartz plate is covered with a 1 nm chromium layer on both sides to avoid charging while still being transparent for the optical pump laser. The position of the slit is optimized for each given beamline setting by scanning it in the horizontal plane while recording the signals of the bunches for measurement and the background contributions using an avalanche photodiode (APD) and a gated oscilloscope (Figure 1b). The oscilloscope outputs the peak-to-peak values of the corresponding bunches in the fill pattern (see Figure 1c–e). The best position of the slit for separating the PPRE from the singlebuch background is given by the position of the maximum of a *Figure of Merit* given by $\frac{{\left(PPRE\right)}^{2}}{Singlebunch}$. The Figure of Merit is the product of the intensity of the bunch of interest and the separation ratio which represents the best compromise between the suppression of background radiation and the transmitted radiation of the PPRE/slicing bunch. For Femtoslicing the signal to optimize is that of the sliced pulse against the background created by the halo of the sliced bunches (see Figure 1e). The halo is an inherent result of the slicing process and is further explained in [10,14].

### 2.2. The Synchronized Optical Laser System for Optical Excitation

**Figure 2:**
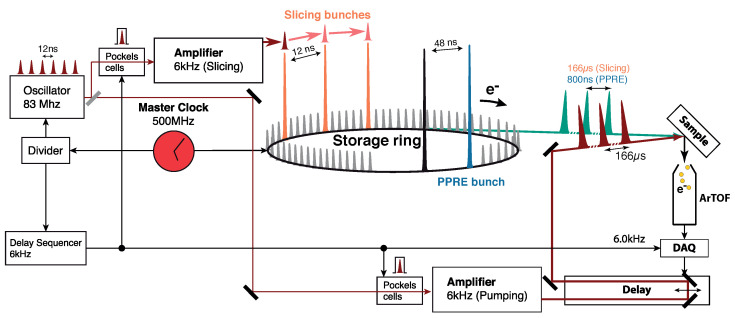
Trigger scheme for a time-resolved photoemission experiment at the FemtoSpeX facility at BESSY II. Two Ti:sapphire amplifiers (Legend Elite Duo, COHERENT), which are seeded by the same oscillator (Micra, COHERENT), are used to drive the slicing process and to pump the sample. The oscillator, running at 83.3 MHz ($\hat{=}$12 ns), is phase-locked to the master clock of the storage ring. Both lasers are running at 6 kHz ($\hat{=}$166 μs). For slicing or *femtosecond* measurements, a delay sequencer is used which advances the trigger for each 6 kHz trigger by 12 ns to reduce the halo background and improve slicing efficiency. For PPRE or low-α measurements, the delay sequencer is bypassed and the pump laser is synchronized to the PPRE pulse on the other side of the fill pattern. Further information on the trigger scheme and the realization can be found in [10,14]. Adapted from Ref. [10].

For optical excitation (pump) we used a Ti:Sapphire amplifier (Legend Elite Duo, COHERENT). This amplifier is driven by the same oscillator (Micra, COHERENT, 800 nm) as the laser which is used to drive the femtoslicing process [10]. The oscillator, running at 83.3 MHz ($\hat{=}$12 ns), is phase-locked to the master clock of the storage ring. The overall trigger scheme is presented in Figure 2. The amplifier generates an energy per pulse of up to ∼1.8 mJ at a maximum frequency of 6 kHz at a center wavelength of 800 nm (FWHM ∼45 fs). Frequency doubling (400 nm, FWHM ∼70 fs) using a Beta Barium Borate (BBO) crystal is available. Additionally, an optical parametric amplifier (OPA) is available, which can deliver laser pulses in the spectral range from about 250 nm to about 30 μm.

The laser fluence using the fundamental and the 2nd harmonic can be adjusted by varying the polarisation of the laser beam in front of two thin film polarizers (LAYERTEC, 400 nm, 56°, R_*s*_ > 99.8%, R_*p*_ < 2%, 800 nm, 65°, R_*s*_ > 99.8%, R_*p*_ < 2%). We use additional reflective UV-grade ND filters to further reduce the fluence (Thorlabs). The fluence is measured in front of the incoupling optics after a metallic mirror with known transmission (LAYERTEC enhanced aluminium with a protective coating, R >80% 200–800 nm, T <4% 200–800 nm) using a photodiode and an oscilloscope. This approach will in the future enable the shot-to-shot acquisition and thus correlation of the laser fluence with time-of-flight spectra. The photodiode can be calibrated using a power meter (Gentec UP19K-15S-W5-D0 and XLP12-3S-H2-D0). The incoupling optics consist of a concave and convex lens with AR coating for focussing the laser on the sample, two metallic mirrors for adapting the beam height (Newport Corporation protected silver or enhanced aluminium depending on the pump wavelength) and a window flange. The delay of the laser pulses relative to the synchrotron radiation pulses is regulated by an optical delay stage. This stage allows a delay range of up to 4.4 ns with femtosecond resolution (Newport DL325, MIM 0.5 fs). The spatial overlap is established with a piezo-driven mirror stage located at the bottom of the periscope for beam incoupling (Newport Corporation Picomotor 8853).

Since the laser system is running at 6 kHz and the synchrotron pulses arrive at a repetition rate of 1.25 MHz in PPRE mode, we can efficiently track the decay of the optically excited system in 800 ns steps up to a timescale of about 166 μs (207 round trips of the X-rays) with a single measurement at a fixed optical delay stage. We are therefore able to study hundreds of electron spectra over several orders of magnitude in time delay (fs to μs). Moreover, the laser timing can be shifted electronically in steps of 12 ns, spanning the delay range from 12 ns up to 800 ns. Finally, the delay range below 12 ns can be covered by shifting the synchronisation of the phase lock continuously.

The combination of the ∼120 fs long X-ray pulses and the ∼70 fs long laser pulses leads to a temporal resolution of ∼140 fs. Using the PPRE mode the temporal resolution is dominated by the pulse length of the X-ray pulses of 50 ps.

### 2.3. The SurfaceDynamics Instrument

The endstation can be separated into five different vacuum sections with individual capabilities. The main chamber is equipped with a high-transmission electron spectrometer based on the time-of-flight principle. The typical resolving power of the instrument is about 3000 for low kinetic energies and about higher than 1000 for the high kinetic energies [7]. This opens the way towards chemical state selective and time-resolved X-ray photoelectron diffraction. It is also equipped with the slit described above to block away the multibunch background and isolate the single-photon pulses. An avalanche photon diode (APD) can be used for optimizing the bunch contrast as well as to perform transmission spectroscopy. The vacuum is on the order of about $6\times 10^{-11}$ mbar, achieved by a combination of conventional turbo molecular pumps (TMP), an ion getter pump (IGP) and a large non-evaporable getter pump (NEG). The top preparation chamber is equipped with a quadrupole mass spectrometer (Pfeiffer Vacuum) and a sputter gun (SPECS Group IQE 11/35). Evaporators can be installed on the two remaining free ports. Both, the top preparation chamber and the main chamber, share the same sample manipulator. The sample can be cooled to about 30 K using LHe thanks to a standard ST-400 cryostat manufactured by Janis. The sample stage itself is equipped with an electron bombardment heating system which can heat the sample to temperatures >2000 K in a few seconds. The endstation has a second preparation chamber equipped with its own sputter gun (SPECS Group IQE 11/35), an LEED system with integrated Auger Electron Spectroscopy capabilities (OCI Vacuum Microengineering Inc. BDL800IR, London, ON, Canada) and a dual cell cluster evaporation source (Dr. Eberl MBE-Komponenten GmbH DCS 40-2x-1-14-2S). Typical evaporators are Au, Ag, Bi, Ga, In, organics, etc. and the operating temperature of the source is in the range of 50–1400 °C. Additionally, a quartz crystal monitor for measuring deposited film thicknesses is available (Inficon STM-2). The base pressure of the preparation chamber is better than $2\times 10^{-10}$ mbar. Both sputter guns are connected to the same gas dosing system with a base pressure better than $5\times 10^{-10}$ mbar and four connections for gases. The manipulator in the preparation chamber can be cooled to LN_2_ temperatures by filling liquid nitrogen into a reservoir. The sample stage is again equipped with an electron bombardment heating system with the same capabilities as in the main/top preparation chamber. A load-lock is used to introduce samples with a pump-down time of about 30 min. The samples are mounted on Omicron-style flag sample holders and can be distributed between the chambers using a radial sample distribution chamber (PreVac).

### 2.4. Measurements Settings and Samples

We now showcase results obtained using the well-known sample system TaS_2_ to demonstrate the capabilities of the setup. The focus is mainly on the time-resolved capabilities but we also highlight XPD results exploiting the large acceptance angle and the high angular resolution.

We used 1T-TaS_2_ samples that were grown by iodine vapour transport [15] by the Rossnagel group (Christian-Albrechts-Universität zu Kiel). Although the growth process is well understood, a natural variation in the measurements presented in the following sections is inevitable, since we also used samples from different growth periods. These variations lead, for example, to different CDW splittings and collapse upon pumping.

In the experiments presented here, the monochromator [10] was operated with a 150 L/mm grating at two distinct photon energies of 200 eV and 250 eV. The exit slit was set to 100 μm resulting in a beamline resolution of about 210 meV at 200 eV and 280 meV at 250 eV respectively. The ArTOF was configured to a mode having an accepting angle of 56° and an energy window 4% of the kinetic energy of the photoelectrons at the core level of interest. With this setting the typical instrument resolution is better than about 150 meV for kinetic energies below 250 eV [7]. We used a wavelength of 800 nm as a pump and the laser fluence impinging on the sample was about 1.5 mJ/cm^2^ for the measurements using the PPRE and about 0.1 mJ/cm^2^ for the measurements using slicing.

Static measurements were carried out by using the PPRE bunch at a repetition rate of 1.25 MHz. The time-resolved studies were done in picosecond mode by using the PPRE bunch and in femtosecond mode by using the slicing bunches as the probe pulses. Both time-resolved modes give us access to unpumped reference pulses from the X-ray bunch one revolution before the pump pulse arrives.

## 3. Results and Discussion

### 3.1. Time Resolved X-ray Photoelectron Spectroscopy with Different Time Scales at 1T-TaS_2_

1T-TaS_2_ has a rich phase diagram consisting, at ambient pressures and decreasing temperatures, of an incommensurate Charge Density Wave (ICCDW) phase, a nearly commensurate CDW (NCCDW) phase and a commensurate CDW (CCDW) phase [16,17] and, at elevated pressures, even metallic and superconducting phases [18].

**Figure 3:**
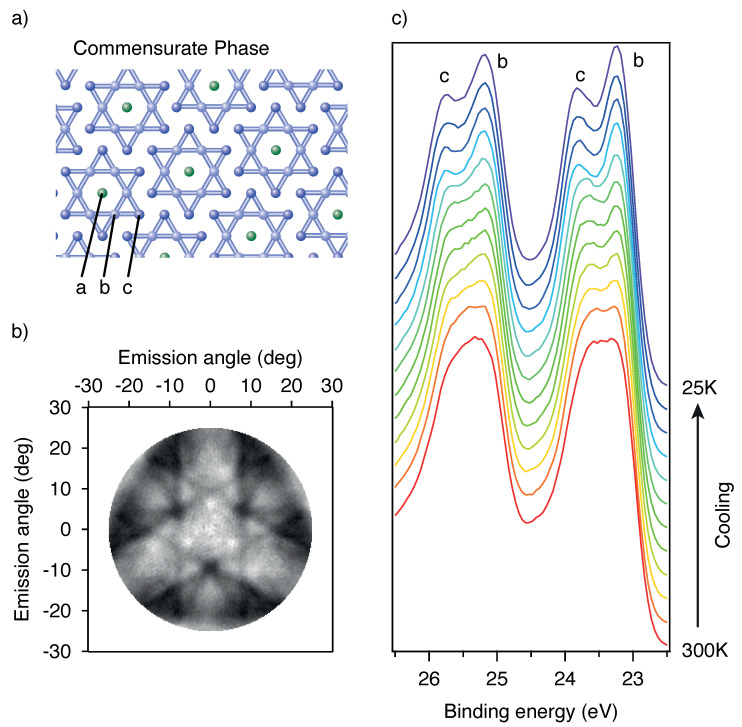
X-ray photoelectron spectroscopy and diffraction at 1T-$TaS_{2}$. Panel (**a**) shows the Star-of-David arrangement of the Ta atoms in the CCDW phase with the central atom (**a**), the nearest neighbour atoms (**b**) and the next-nearest neighbour atoms (**c**). Panel (**c**) shows, as a function of temperature, that 1T-$TaS_{2}$ undergoes a phase transition (NCCDW → CCDW) to a charge-separated star of David reconstruction at low temperature panel (**a**), associated with chemically shifted Ta 4f states. Panel (**b**) shows that the wide angle lens ArTOF of the SurfaceDynamics endstation is able to detect next to the X-ray photoelectron spectra a full set of X-ray diffraction pattern with a collection angle of 56° at an energy window of 4%.

The main structure of TaS^2^ is made up of sheets of S-Ta-S atoms in a CDW ordering close to a $\sqrt{13}\;\times \sqrt{13}$ superstructure. Cooling from the incommensurate to the commensurate phase results in a rotation of the superstructure from 0° to 13.9° [17]. The CCDW is accompanied by a periodic lattice distortion (PLD) which creates a ‘Star-of-David’ structure (see Figure 3a). The Ta atoms create a 13-atom cluster where the nearest and next-nearest neighbour Ta atoms move towards the central Ta atom. This leads to three types of chemical surroundings for the Ta atoms and thus to a strong ESCA splitting of the Ta $4f_{5/2}$ and $4f_{7/2}$ lines (see Figure 3c). The amount of this splitting can be used to quantify the amplitude of the CDW/PLD and to follow its dynamics upon laser excitation.

The intensity ratio of the three types of Ta atoms should be 6:6:1 from stoichiometry. However, we observe no third peak from the ‘a’ atoms and a deviation from the naive stochiometry intensity ratio, between ‘b’ and ‘c’ atoms as already reported in the literature [19]. In Figure 3c we also show the Ta 4f spectrum during the cooling of the sample from room temperature to 20 K in quasi-thermal equilibrium. The increasing ESCA separation between components ‘b’ and ‘c’ of the Ta 4f lines reflects the emerging CDW/PLD, as discussed in Ref. [4]. We also measured the X-ray photoelectron diffraction (XPD) pattern created by the Ta4f electrons at a photon energy of 840 eV. The symmetrized diffraction pattern is a result of integrating the spectra within the kinetic energy range from 811 eV to 815 eV and is depicted in Figure 3b. One main Kikuchi band is clearly visible as well as the 3-fold symmetry [20]. Although the obtained pattern is only showing a $\pm 28^{{}^{\circ}}$ region of the full pattern, one can see the characteristic features of a chalcogen terminated surface [20].

The Ta 4f trPES of TaS_2_ has been studied earlier, for example at the FLASH free-electron facility in Hamburg [4,16] and using an HHG pulsed laser system [21]. The experiment described in [4,16] used 156 eV X-Ray photons and mitigated the FEL-induced space charge effects by moving the interaction point out of the beamline focus to decrease the photon density impinging on the sample. Moreover, the timing jitter was on the order of 700 fs. The pulse length of the optical pump laser was about 120 fs (FWHM). The authors of [21] used a laser-based HHG source which is inherently free of timing jitter. However, they used a photon energy of 60 eV and pump laser energy of 3.15 eV with a pulse length of about 170 fs (FWHM). In our femtosecond setup, the overall time resolution was about 140 fs [10] consisting of the pulse length of the 800 nm pump (∼70 fs) and of the X-Ray probe (∼120 fs). To summarize, in FEL based experiments one has to carefully take care of the FEL induced space-charge and of the timing jitter, whereas in an HHG-based experiment one has limited access to deeper core levels due to the low photon-energy cut-off. In our case, the experiment is jitter-free, and we have access to deeper core-levels, however, the intensity is rather low, and we have to deal with longer integration times.

**Figure 4:**
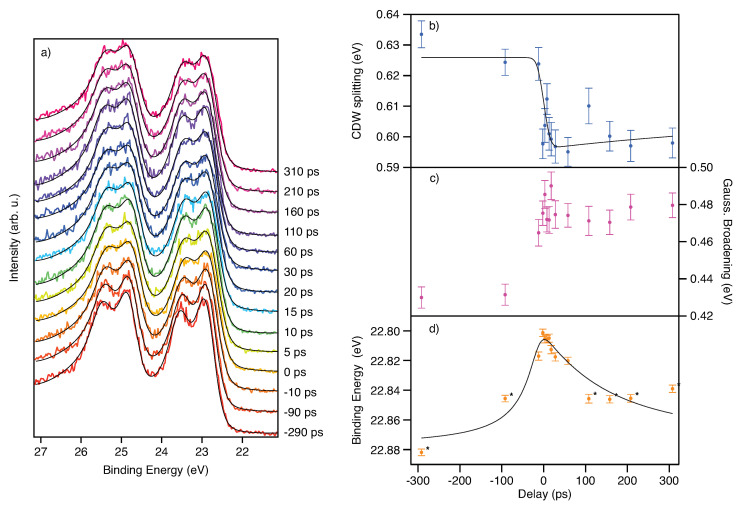
Picosecond pump-probe XPS Data of TaS_2_. Panel (**a**) shows the time-resolved spectra of the Ta 4f lines acquired at different pump-probe delays. The delay values have been adjusted to match the zero delay resulting from the fit. In the panels (**b**–**d**) we show the delay traces of the CDW splitting, the Gaussian broadening and the binding energy position, respectively. The data points marked with the asterisk (*) have ∼5 times less statistical weight (see text). The spectra have been acquired using a photon energy of 200 eV. Black lines represent the fit results.

**Figure 5:**
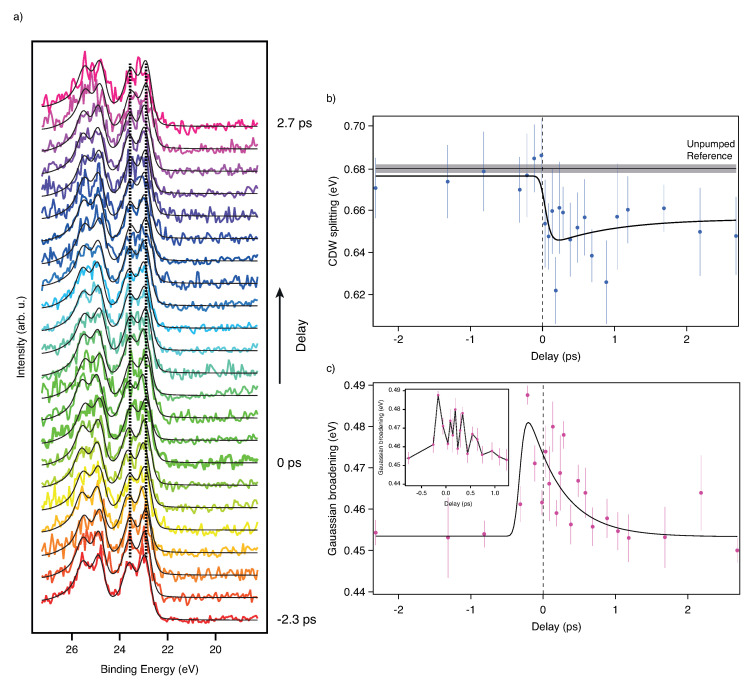
Femtosecond pump-probe XPS Data of TaS_2_. Panel (**a**) shows the photoelectron spectra of the Ta 4f lines acquired with X-ray pulses delivered by the slicing source. The spectrum corresponding to the extracted temporal overlap of the X-ray pulses with the laser pulse is marked with 0 ps. Additionally, the fits to the data are presented (thin black lines). The vertical dashed lines show the peak positions of the reference spectrum. Panel (**b**) shows the dynamics of the CDW splitting and panel (**c**) the dynamics of the Gaussian broadening of the peaks. The inset in panel (**b**) shows the region close to the delay zero.

We now discuss the time-resolved results obtained using the pump-probe technique. As a first step, we used the PPRE operation mode and focussed on the picosecond dynamics of the CDW/PLD upon laser excitation. In Figure 4a, we show spectra acquired for different pump-probe delay times. For the negative delays (red to orange) well split Ta 4f lines are visible due to the CDW/PLD. After zero delay, the collapse of the CDW/PLD is qualitatively visible, even in the raw spectra (three raw spectra with fits and residuals are presented in the Supplementary Material (Figures S1–S3). For further analysis, we have fitted two Doniac-Sunjič pairs. The constant background (emerging from multibunch photon background), the linear background, the CDW splitting, the overall Gaussian broadening and the position were free parameters in the fit. The other parameters were obtained from the fit to the unpumped MHz reference spectrum. For this step, the spin-orbit (SO) splitting and the asymmetry parameters in the Donjiac–Sunjič lineshape were first taken from literature [22] and then refined using the unpumped reference spectrum. We extracted, using the reference spectrum, the values reported in Table 1:

The fitting errors are derived from the fitting routine of IGOR Pro 7 (Wavemetrics). We used the Poisson statistic to estimate the errors for each energy bin in all spectra. The value of 74 meV for the Ta 4f natural core-hole lifetime broadening is consistent with literature values [23]. The intensity ratio of the spin-orbit split peaks is not 3:4 as expected for 4f lines. The asymmetries are about 0.057 higher than reported in [22]. The delay-dependent results of the spectral fits are presented in panels (b–d) of Figure 4. For the fit of the dynamics of the CDW splitting we used a double exponential decay model convoluted with a Gaussian representing the time resolution. Again, we fixed as many parameters as possible. The SO splitting was fixed to 1.912 eV, the natural linewidth to 74 meV and the asymmetries to 0.289 for site b and 0.360 for site c. The initial decay was fixed to 0.1 ps which is well within the literature range from <50 fs to 700 fs [4,16,21,24]. In our case the initial decay is solely governed by the time resolution. The value for the time constant of the recovery was fixed to 655 ps [4]. Since we have only measured up to a delay of about 300 ps, we were not able to let this parameter vary. The raw delay zero and the temporal resolution have been extracted by a space-charge model, as described by multiple authors in [4,25,26,27,28]. The model is extended with a convolution by a Gaussian function representing the width of the probe pulse. The width of this function is a free parameter in the fit and leads to a smoothing of the potential at zero delay. Also, the zero delay point is a free parameter, as are the number of generated laser photons and their average kinetic energy. The fit was a global fit, meaning that we fitted the CDW dynamics and the space-charge shift (SCS) at the same time and linked the time zero and the resolution of both fits. Since we do not have many data points for large negative and positive delays, these points have been assigned ∼5 times less statistical weight in the SCS model. Using this model we extracted the parameters in Table 2:

We now turn to the results obtained using fs X-ray slicing pulses as a probe. In Figure 5a we show the spectra obtained for various delay points. Subtraction of the halo background was not necessary. However, as shown in Figure 1d, there is always a halo contribution. Evaluating both, the slicing and halo contribution based on the total acquired counts in the energy window of the spectrometer, we find a slice-to-halo ratio of about 2:1. The halo is a result of incomplete equilibration of the excited electrons in the electron bunch after one turn in the storage ring. More details can be found in Holldack et al. and Schick et al. and references therein [10,14]. The halo results in an about 100 ps long light pulse on the sample. Due to the long pulse, the ultrafast dynamics are averaged when probed by the halo pulse while at the same time, the fraction of the sliced pulse gives rise to ultrafast dynamics on top of this average background. Since we acquire every trigger for each sliced and pumped pulse also the following 206 revolutions, we have access to the halo contribution just one revolution before the next trigger event (see Figure 1a. As a next step we have fitted again Donjiac–Sunjič lineshapes to the spectra (see Figure 5a and Figures S4–S6 in the Supplementary Materials). For fitting we have kept the Lorentzian line width, the SO splitting, the SO ratio, the CDW ratio and the asymmetries fixed to the fit parameters as derived from reference spectra. The constant background, the peak position, the CDW splitting, the peak height and the Gaussian broadening were kept as free fit parameters. The process of melting of the CDW can be seen in Figure 5b and is taking place in less than 1 ps, which is consistent with the observations made in Ref. [4].

In Figure 5c we have plotted the Gaussian broadening of the Ta 4f lines versus the delay time. A constant broadening can be observed for negative delays. At the temporal overlap, the broadening increases abruptly and shrinks again to the value observed for negative delays within about 1 ps. This is in accordance with the observation made by Ishizaka et al. [21]. Unfortunately, due to undersampling, we cannot resolve the oscillation the last authors observed in their experiment. However, one can note that our data is not aligned with a simple exponential decay model. Rather, the data points exhibit an extensive spread, larger than the errors from the fit. This could hint towards the oscillatory behaviour, although we cannot resolve it directly. We have fitted both the CDW dynamics and the dynamics of the broadening with the same double exponential model function as in the picosecond CDW data. The fit results are presented in Table 3:

Although the model function does capture the dynamics, there are peculiarities to be addressed. First of all, the reduced $\chi^{2}$ in the CDW fit is below one, which implicates overfitting. It was not possible to capture the time constant for the fast decay due to a lack of delay points in this region. Also, the error of the time constant of the slow recovery is much larger than its value. The uncertainty of the fit results is solely governed by the statistics of the measurement.

For the fit of the broadening, we get a reduced $\chi^{2}$ above one, which indicates underfitting. It seems that here the model function does not quite capture the data points. Again it was impossible to fit the fast rise of the broadening due to too few data points in this region. However, the errors of the other fit parameters are reasonable. The reason for underfitting could be assigned to the aforementioned oscillations which can appear on the broadening and which are not part of the model function.

It can be seen that there is a shift of the $t_{0}$ between the broadening and the CDW dynamics of about 0.36 ps ± 0.08 ps. A similar effect was observed by the authors of [21].

However, with the present data set, no further conclusions can be drawn. This is simply because doing a femtosecond time-resolved photoemission experiment at a slicing source is an extremely time-consuming effort, due to the low count rate. The acquisition time per delay step was about 50 min for this combination of core-levels and photon energy. Improving the source in terms of photon flux would definitely mitigate these issues. Additionally, the aforementioned sample variations also extend to the maximum achievable CDW splitting and the effect upon pumping with an optical laser. For our sample set, it was not possible to find a spot which has high CDW splitting and a high contrast upon pumping. The value of the collapse of the CDW in [4,16] is about 150 meV and about 100 meV in [21]. We find values below 50 meV. The reason is that the samples were with a size of about 4 mm^2^ rather small and in-vacuum cleaving rarely resulted in a flat surface. However, the stability of the slicing source allowed us to observe the collapse of the CDW upon pumping although the effect was rather small as compared to previous published results.

### 3.2. Towards Time-Resolved X-ray Photoelectron Diffraction at TaS_2_

We have so far dealt with the time-resolved aspects of the experimental scheme, to which we now add the aspect of simultaneous angular resolved Photoelectron Diffraction. This is inherently possible by the large acceptance angle of the ArTOF analyser. From a scientific point of view, the aspect of simultaneous acquisition of chemical ESCA and structural XPD information from the same sample site is a major advantage. To benchmark such performance, we conducted an X-ray photoelectron diffraction (XPD) experiment using again 1T-TaS_2_ as our model functional material, which is well understood, and we can compare to published results [20]. As shown in Figure 3b), the full photoelectron diffraction pattern is readily observed, with the photoemission spectrum being also accessible by integration over the emission angles. Technically, we have been running this proof of concept approach at high kinetic electron energies of 815 eV which directly influences the spectral resolution which was on the order of 900 meV [7]. The integration time, using the single bunch operation mode, for this static XPD image was about 15 min currently limited by the lacking multi-hit capability of our detector. We integrated over the b and c sites (see Figure 3a,c) since the splitting was below the energy resolution. However, adsorbed molecules on single crystals can lead to large ESCA shifts. For these cases state selective XPD is advantageous. With improved detector performance, time-resolved XPD is also possible.

## 4. Conclusions

With the SurfaceDynamics instrument, we have established the feasibility of combined chemical and structural dynamics investigations on functional materials by time-resolved electron spectroscopy for chemical analysis and photoelectron diffraction. The up to 60° acceptance angle of the angle-resolved time of flight electron spectrometer gives combined temporal and angular resolution with spectral detection. We conducted these proof of concept studies at the FEMTOSPEX facility of the BESSY II storage ring, where picosecond Synchrotron pulses can be extracted, but also shorter femtosecond pulses at the price of strongly reduced intensity. Thus, a very large time interval—spanning from the 100 fs timescale (femtoslicing) over a few picoseconds (low-alpha) to tens of picoseconds (regular multibunch) up to a hundred microseconds—can be explored. We showcase these capabilities by presenting the melting of the CDW in cooled 1T-TaS_2_ excited by a femtosecond laser. The rapid evolution of pulsed X-ray sources, in particular, optical laser-driven high harmonic and free-electron laser sources, will make this approach widely applicable. Especially, combining time-resolved structural information from XPD and chemical state information from ESCA in a single experiment at higher photon energies is of interest since the intertwining of structural and electronic dynamics is at the heart of functional materials.

## Figures and Tables

**Table 1:** Fitting results for the unpumped reference spectrum.

Parameter	Value	Std. Dev.
SO Splitting (eV)	1.912	0.001
SO Ratio	0.846	0.002
CDW Ratio	0.591	0.002
Asymmetry site b	0.289	0.001
Asymmetry site c	0.360	0.001

**Table 2:** Fitting results for the global fit of X-ray probe pulse width, delay zero and the CDW dynamics.

Parameter	Value	Std. Dev.
SCS Model		
Probe Pulse width (ps)	29	12
Raw Zero Delay (ps)	−8	2
# of electrons	11,100	1200
Average kin. Energy (eV)	11	5
Laser Spot Size Diameter (μm)	400	fixed
CDW Model		
CDW Splitting unpumped (eV)	0.626	0.003
Amplitude fast decay (eV)	0.030	0.005
Amplitude slow recovery (eV)	−0.01	0.02
Time constant fast decay (ps)	0.1	fixed
Time constant slow recovery (ps)	655	fixed

**Table 3:** Fitting results of the femtosecond CDW and Gaussian broadening dynamics.

Parameter	Value	Std. Dev.
Broadening Dynamics		
Value before t_0_ (eV)	0.453	0.002
Amplitude fast decay (eV)	−0.043	0.006
Amplitude slow recovery (eV)	0.043	0.005
Time constant fast decay (ps)	0.05	fixed
Time constant slow recovery (ps)	0.46	0.12
t_0_ position (raw ps)	−210.04	0.02
Temporal resolution (ps)	0.12	fixed
reduced $\chi^{2}$	3.79	n.a.
CDW Dynamics		
Value before t_0_ (eV)	0.676	0.007
Amplitude fast Decay (eV)	0.034	0.019
Amplitude slow recovery (eV)	−0.014	0.017
Time constant fast decay (ps)	0.05	fixed
Time constant slow recovery (ps)	0.9	4.2
t_0_ position (raw ps)	−209.68	0.08
Temporal resolution (ps)	0.12	fixed
reduced $\chi^{2}$	0.53	n.a.

## Data Availability

The data presented in this study are available on request from the corresponding author. The data are not publicly available due to the large size of the raw data.

## Supplementary Materials

The following supporting information can be downloaded at: https://www.mdpi.com/article/10.3390/molecules27248833/s1, Figures S1–S3: Raw data of Ta4f peaks (PPRE); Figures S4–S6: Raw data of Ta4f peaks (Slicing).

## Abbreviations

The following abbreviations are used in this manuscript:

ESCA	Electron Spectroscopy for Chemical Analysis
XPD	X-ray Photo Electron Diffraction
ArTOF	Angle resolved Time-of-Flight
FEL	Free-Electron Laser
VUV	Vacuum Ultra-Violet
SASE	Self-Amplified Spontaneous Emission
HHG	Higher Harmonics Generation
TaS^2^	Tantalumdisulphide
PGM	Plane Grating Monochromator
PPRE	Pulse Picking by Resonant Excitation
TRIBs	Transverse Island Buckets
FWHM	Full Width at Half Maximum
APD	Avalanche Photo Diode
MB	Multibunch
CS	Camshaft (bunch)
BBO	Beta Barium Borate
OPA	Optical Paramteric Amplifier
UV	Ultra-Violet
ND	Neutral Density
AR	Anti Reflective
MIM	Minimum Incremental Motion
TMP	Turbo Molecular Pump
IGP	Ion Getter Pump
NEG	Non-Evaporable Getter Pump
LHe	Liquid Helium
LEED	Low-Energy Electron Diffraction
LN_2_	Liquid Nitrogen
CDW	Charge Density Wave
ICCDW	Incommensurate Charge Density Wave
NCCDW	Nearly Commensurate Charge Density Wave
CCDW	Commensurate Charge Density Wave
PLD	Periodic Lattice Distortion
XPS	X-ray Photoelectron Spectroscopy
SO	Spin-Orbit
trPES	Time-Resolved Photoemission Spectroscopy
FLASH	Free-Electron Laser in Hamburg
HHG	Higher Harmonic Generation
SCS	Space-Charge Shift
