# Supplementary material for: Time and Angle-Resolved Time-of-Flight Electron Spectroscopy for Functional Materials Science
Source: Molecules. 2022 Dec 13;27(24):8833. doi: 10.3390/molecules27248833 (PMC9787937; doi:10.3390/molecules27248833)
Supplement: Supplementary file 1 [file molecules-27-08833-s001.zip › molecules-2038743-Supplementary Material.pdf]

molecules

Article

Supplementary Material: Time and Angle Resolved Time-of-Flight Electron Spectroscopy for Functional Materials Science

Nomi Lucia Ada Nathalie Sorgenfrei [1,*], Erika Giangrisostomi [1], Danilo Kühn [1], Ruslan Ovsyannikov [1] and Alexander Föhlisch [1,2]

1 Institut für Methoden und Instrumentierung der Forschung mit Synchrotronstrahlung, Helmholtz-Zentrum Berlin für Materialien und Energie GmbH, Helmholtz-Zentrum Berlin GmbH, Albert-Einstein-Str. 15, 12489 Berlin, Germany
2 Institut für Physik und Astronomie, Universität Potsdam, Karl-Liebknecht-Stra§e 24/25, 14476 Potsdam, Germany
* Correspondence: nomi.sorgenfrei@helmholtz-berlin.de; Tel.: +49-30-8062-18036

Keywords: photoelectron spectroscopy, surface science, time-resolved, ultrafast, instrumentation, dichalcogenides, phase transition

check for updates

Citation: Sorgenfrei, N. L. A. N.; Giangrisostomi, E.; Kühn, D., Ovsyannikov, R.; Föhlisch, A. Supplementary Material: Time and Angle Resolved Time-of-Flight Electron Spectroscopy for Functional Materials Science. *Molecules* **2022**, *27*, 8833. https://doi.org/10.3390/molecules27248833

Academic Editor: Frantisek Hartl

Received: 1 November 2022
Accepted: 10 December 2022
Published: 13 December 2022

Publisher's Note: MDPI stays neutral with regard to jurisdictional claims in published maps and institutional affiliations.

Copyright: © 2022 by the authors. Licensee MDPI, Basel, Switzerland. This article is an open access article distributed under the terms and conditions of the Creative Commons Attribution (CC BY) license (https://creativecommons.org/licenses/by/4.0/).

In the following we show raw data together with the corresponding fits and residuals of 3 representative measurements of different pump-probe delays for the picosecond (PPRE) mode (Figures S1–S3) and for the femtosecond slicing mode (Figures S4–S6):

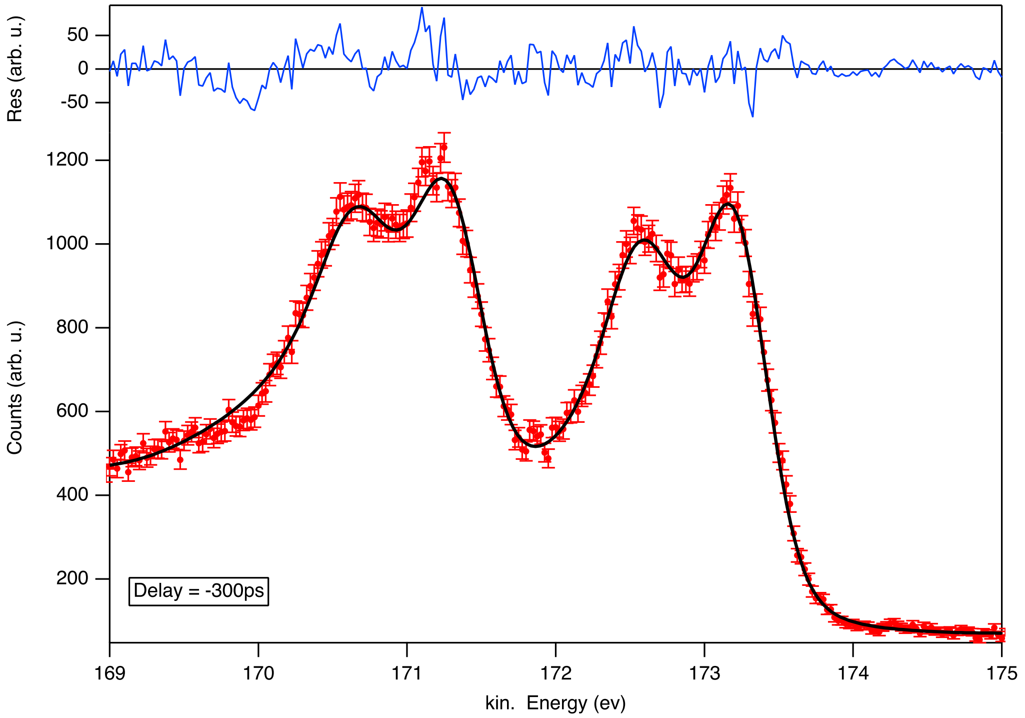

Figure S1. Raw data of the Ta4f peaks measured at 200eV photon energy using the PPRE mode at a delay of -300ps (red markers, error bars are derived using counting statistics). The black line shows the fit result and the blue line the residual.

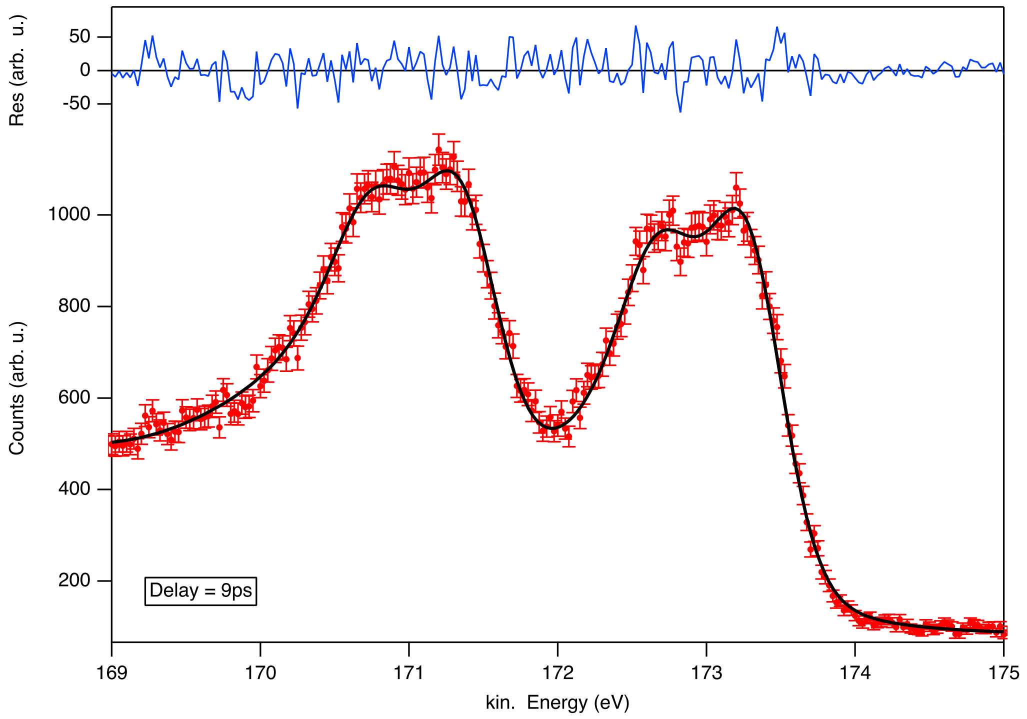

Figure S2. Raw data of the Ta4f peaks measured at 200eV photon energy using the PPRE mode at a delay of 9ps (red markers, error bars are derived using counting statistics). The black line shows the fit result and the blue line the residual.

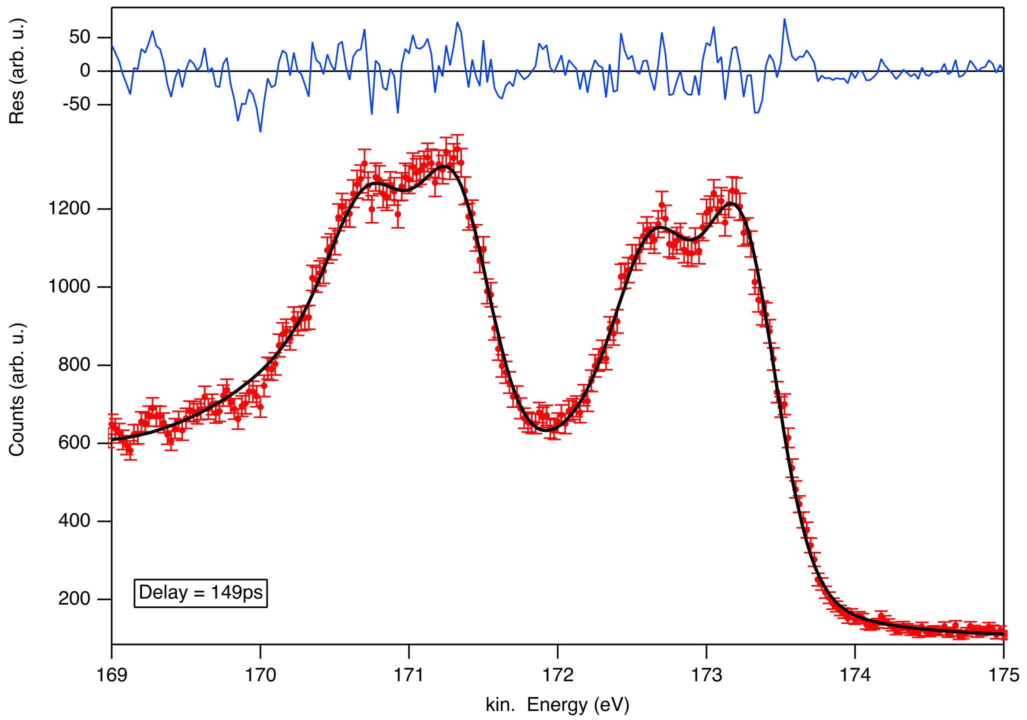

Figure S3. Raw data of the Ta4f peaks measured at 200eV photon energy using the PPRE mode at a delay of 149ps (red markers, error bars are derived using counting statistics). The black line shows the fit result and the blue line the residual.

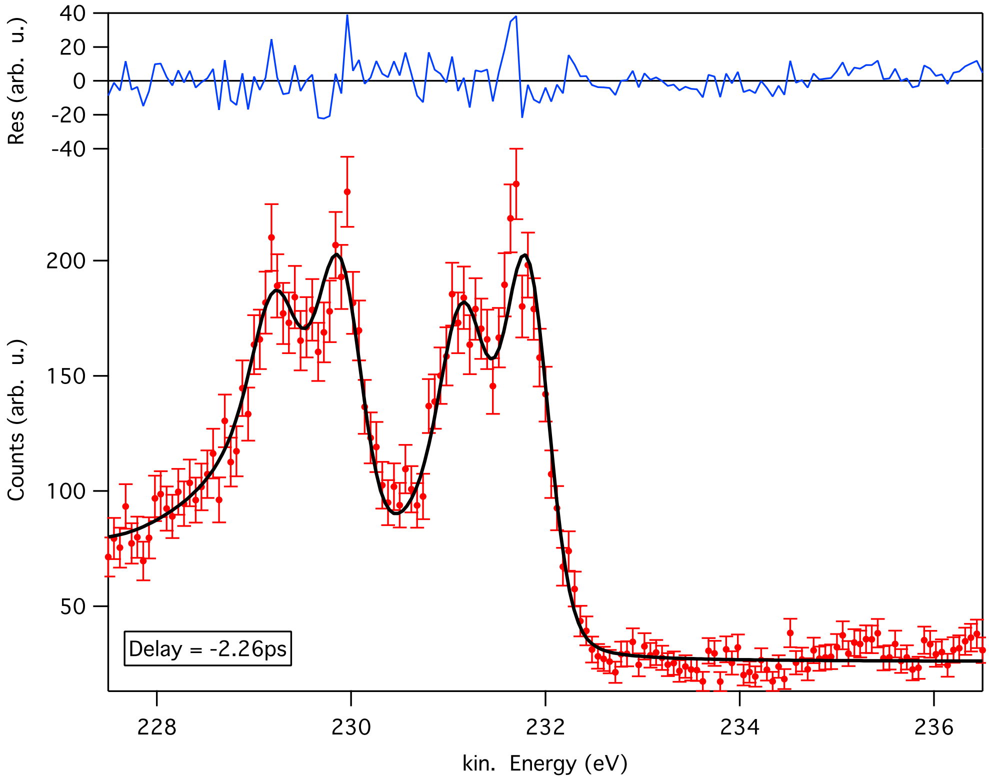

Figure S4. Raw data of the Ta4f peaks measured at 260eV photon energy using femtoslicing at a delay of -2.26ps (red markers, error bars are derived using counting statistics). The black line shows the fit result and the blue line the residual.

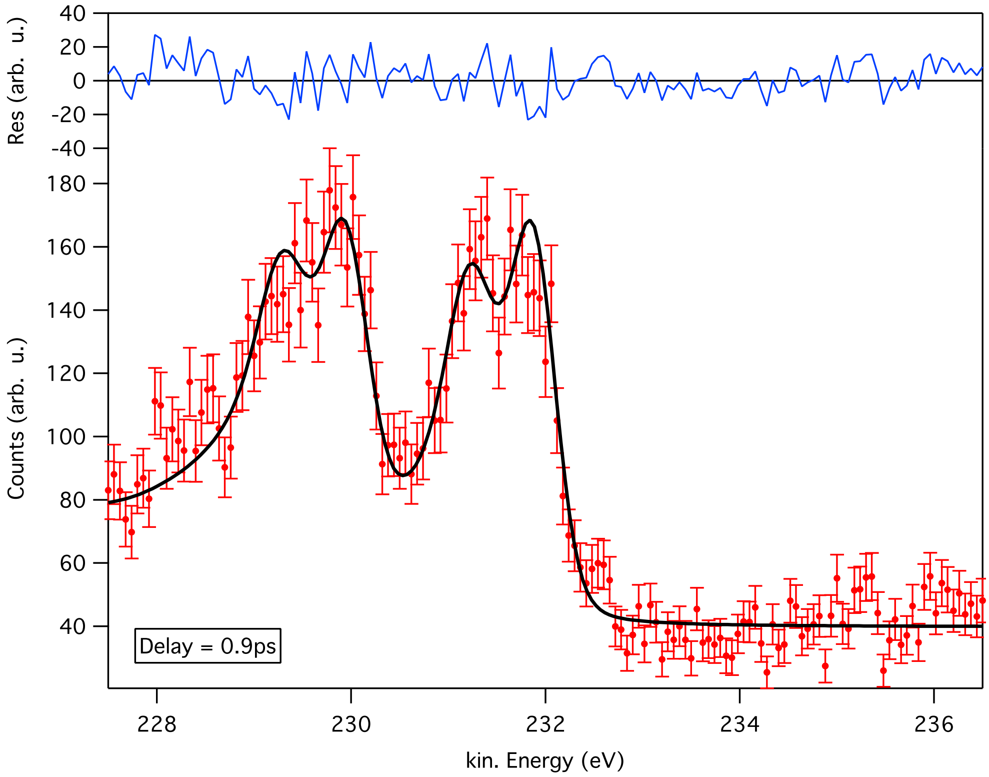

Figure S5. Raw data of the Ta4f peaks measured at 260eV photon energy using femtoslicing at a delay of 0.9ps (red markers, error bars are derived using counting statistics). The black line shows the fit result and the blue line the residual.

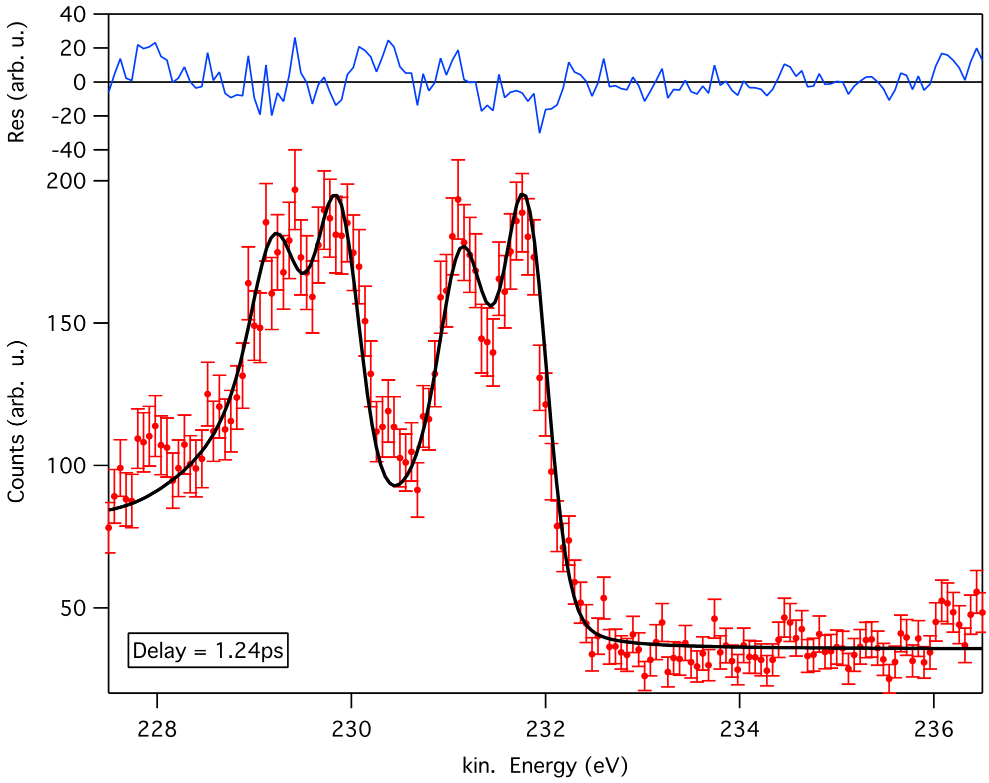

Figure S6. Raw data of the Ta4f peaks measured at 260eV photon energy using femtoslicing at a delay of 1.24ps (red markers, error bars are derived using counting statistics). The black line shows the fit result and the blue line the residual.
